# Practical guide to exocrine pancreatic insufficiency – Breaking the myths

**DOI:** 10.1186/s12916-017-0783-y

**Published:** 2017-02-10

**Authors:** Maarten R. Struyvenberg, Camilia R. Martin, Steven D. Freedman

**Affiliations:** Beth Israel Deaconess Medical Center, 330 Brookline Ave., Dana 501, Boston, MA 02215 USA

**Keywords:** Exocrine pancreatic insufficiency, Lipase, Steatorrhea, Fat malabsorption, Pancreatic enzyme replacement therapy, Pancreatic enzymes

## Abstract

**Background:**

Exocrine pancreatic insufficiency (EPI) is characterized by a deficiency of exocrine pancreatic enzymes, resulting in malabsorption. Numerous conditions account for the etiology of EPI, with the most common being diseases of the pancreatic parenchyma including chronic pancreatitis, cystic fibrosis, and a history of extensive necrotizing acute pancreatitis. Treatment for EPI includes dietary management, lifestyle changes (i.e., decrease in alcohol consumption and smoking cessation), and pancreatic enzyme replacement therapy.

**Discussion:**

Many diagnostic tests are available to diagnose EPI, however, the criteria of choice remain unclear and the causes for a false-positive test are not yet understood. Despite multiple studies on the treatment of EPI using exogenous pancreatic enzymes, there remains confusion amongst medical practitioners with regard to the best approach to diagnose EPI, as well as dosing and administration of pancreatic enzymes.

**Summary:**

Appropriate use of diagnostics and treatment approaches using pancreatic enzymes in EPI is essential for patients. This opinion piece aims to address the existing myths, remove the current confusion, and function as a practical guide to the diagnosis and treatment of EPI.

## Background

Exocrine pancreatic insufficiency (EPI) is defined by a deficiency of exocrine pancreatic enzymes resulting in an inability to maintain normal digestion. This inadequate digestion with nutrient and, especially, fat malabsorption occurs when intraduodenal levels of lipase fall below 5–10% of normal enzyme output [1], leading to pancreatic steatorrhea, weight loss, and a potential decrease in quality of life [2–5]. Furthermore, in EPI, due to cystic fibrosis (CF) or chronic pancreatitis, there is decreased bicarbonate output causing a lower intestinal pH, which precipitates bile salt acids and impairs micelle formation of fats [6, 7]. Fat maldigestion is compounded by decreased pancreatic secretion of lipase and colipase, further dampening hydrolysis of intraluminal fat.

A myriad of tests for EPI have been developed over the years, however, the choice of which to use remains unclear as does the understanding of causes for a false-positive test. In addition, there is significant confusion over dosing and administration of pancreatic enzymes. This opinion piece aims to address the existing myths, remove the current confusion, and function as a practical guide to the diagnosis and treatment of EPI.

## Discussion

*Myth: EPI occurs only with more than 90% loss of exocrine pancreatic function*

*Expert opinion: EPI represents a graded response rather than a precise cut-off in pancreatic function and thus patients may benefit from early testing*

A leading cause of EPI is chronic pancreatitis [8]. Other pancreatic causes include a history of extensive necrotizing acute pancreatitis, pancreatic cancer, pancreatic surgery, and CF. Non-pancreatic causes are celiac disease, diabetes mellitus, Crohn’s disease, gastric surgery, short bowel syndrome, and Zollinger–Ellison syndrome [9]. Symptoms of EPI can include steatorrhea (clay-colored, loose, greasy, foul-smelling large stools), abdominal discomfort, bloating, and weight loss. Although floating stools are often thought of being indicative of steatorrhea, they are not; rather sticking to the toilet bowl is a more specific sign.

### Chronic pancreatitis

Chronic pancreatitis is an ongoing inflammatory process with irreversible morphological changes to the pancreas and a gradual loss in pancreatic parenchyma. There are three major groups of mutations that account for chronic pancreatitis (PRSS1, SPINK1, and CFTR). Theories of pathogenesis include oxidative stress, toxic-metabolic derangements, loss of ductal function or obstruction, and necrosis-fibrosis [10].

In chronic pancreatitis, approximately 20% of patients develop EPI over time as a result of progressive loss of acinar cell function [11]. Layer et al. [11] found that the median duration from the onset of symptomatic disease to EPI was significantly longer in early-onset chronic pancreatitis (median age of onset being 19.2 years) than in late-onset idiopathic chronic pancreatitis (median age of 56.2 years) or alcoholic pancreatitis (median age of 13.1 years).

### Cystic fibrosis

CF is an autosomal recessive disorder caused by a mutation of the gene that encodes for a chloride channel called the cystic fibrosis transmembrane conductance regulator (CFTR). In ductal epithelial cells, CFTR is highly expressed and functions to transport fluid and anions into the lumen [12]. Dysfunction of the CFTR gene leads to a decrease in luminal fluid volume and decreased pH, resulting in protein precipitation within the ductal lumen and loss of normal acinar cell function.

EPI is most commonly observed at birth or soon after due to in utero exocrine pancreatic damage. Waters et al. [13] showed that, during newborn screening, 63% of infants with CF are exocrine insufficient and almost 30% of the pancreas-sufficient group will become exocrine insufficient over the next 36 months. Individuals with class IV, V, or VI mutations (less severe CFTR mutations and hence some preserved CFTR function), tend to suffer from EPI later in life [14, 15]. Corey et al. [16] compared 1000 patients from CF clinics in Boston and Toronto and demonstrated that prolonged untreated EPI is associated with a worse long-term outcome and that patients maintained on a high fat diet (100 g per day) with higher doses of exogenous pancreatic enzymes did better than those on a low fat, lower pancreatic enzyme regimen. Overall, pancreatic insufficiency requiring lifelong pancreatic exocrine replacement therapy (PERT) is found in about 85% of CF patients [12].

A rare complication of PERT is seen only in patients with CF on very high doses of enzymes. Fitzsimmons et al. [17] found that, in children with CF, there was a strong correlation between high daily doses of PERT and the development of fibrosing colonopathy. However, this represents a small case series published in *NEJM* in 1997, which were not biopsy proven with very few cases reported since then. Factors related to CF, including thick intestinal secretions, dosing of PERT, and agents in the enteric coating of the pancrelipase preparations may be the precipitating factors causing this complication [18]. Therefore, it has been recommended that, in children and adults with CF, the daily dose should remain below 2500 lipase units/kg of body weight per meal or 10000 lipase units/kg of body weight per day [17, 19].

### Post pancreatic surgery

Factors that contribute to EPI following pancreatic surgery are a decrease in pancreatic tissue volume, extensive denervation following lymph node dissection, and surgically altered anatomy [6]. Conditions such as pancreatic cancer, intraductal papillary mucinous neoplasms, premalignant mucinous cystic lesions, and benign tumors of the pancreas may all lead to EPI via obstruction of the pancreatic duct. The degree of EPI following pancreatic surgery is dependent on the extent of pancreatic resection combined with the degree of residual pancreatic parenchymal function with full manifestation of EPI seen following a total pancreatectomy [20, 21]. The mechanism of EPI in patients undergoing a Whipple procedure may be related to a mistiming of secreted endogenous pancreatic enzymes mixing with chyme.

Large systematic reviews report a 19–80% incidence of EPI following a distal pancreatectomy [22–25]; however, this wide variation may be in part a result of the different diagnostic methods employed [22, 26]. Post-operative incidence of EPI after Whipple surgery is 56–98% [23, 24, 27–29]. In addition, Halloran et al. [28] analyzed 40 patients following resection for pancreatic malignancy and found that EPI was common and sustained after surgery, but was not associated with significant symptoms. These patients with newly developed EPI, however, did have a tendency towards poorer quality of life.

### Celiac disease

Celiac disease is a chronic inflammatory intestinal disorder that may occur in genetically predisposed people triggered by the ingestion of gluten. This disease has a United States and British prevalence of approximately 1% [30, 31]. In celiac disease, although exocrine pancreatic function is intrinsically normal, reduced levels of cholecystokinin release as a result of the duodenal villous atrophy, accounts for impaired gall bladder contraction and reduced exocrine pancreatic secretion [32, 33].

### Diabetes mellitus

The term islet-acinar axis has been used to describe the endocrine-exocrine relationship within the pancreas, whereby there is a vascular and physiologic interaction between these different cell types [34]. Pathophysiologically, diabetes mellitus can predispose to EPI and, conversely, longstanding EPI can be associated with diabetes [35–39]. In diabetes, there are several possible causes which can account for EPI – the lack of the trophic action of insulin (and potentially of glucagon and somatostatin) on acinar cells, autoimmune damage of islet cells, causing destruction of both endocrine and exocrine tissue, and decreased exocrine pancreatic secretion as a complication of diabetic neuropathy [36, 37]. Therefore, the lack of insulin production and the autoimmunity in type I diabetes explain the higher observed prevalence of EPI compared to those with type II diabetes (about 60% vs. 30%) [38]. In addition, a recent article by Soave et al. [39] showed that the lower the immunoreactive trypsinogen levels at birth in newborns with CF, reflecting more severe exocrine pancreatic disease in utero, the earlier in life they developed CF-related endocrine disease (diabetes).

### All infants

Based on the study by Lebenthal and Lee [40] indicating that the duodenal fluid of newborns and infants contained no amylase and negligible lipase at least for the first month of life, all healthy term infants are exocrine pancreatic insufficient. Normally, this is compensated for by amylase and lipase present in breastmilk. However, in formula-fed infants, EPI would be expected. In fact, a recent study by Martin et al. [41] confirmed that formula-fed preterm infants had impaired fatty acid absorption evident through 6 or more weeks postnatal age compared to breastmilk-fed infants, and this is consistent with limited pancreatic lipase production by the pancreas [41]. Thus, all infants, both term and preterm, represent the largest population of individuals with EPI. The clinical implications of developmental pancreatic insufficiency in non-breast-fed infants is unknown, but may play a role in early nutrient deficits in critically ill newborns such as the preterm infant.

### How do we test for exocrine pancreatic insufficiency?

*Myth: Routinely checking for EPI in patients with chronic diarrhea, using fecal elastase, is a useful and effective diagnostic approach with values 100–200 μg/g of stool reflecting mild to moderate pancreatic insufficiency and 100 μg/g of stool indicating severe EPI*

*Expert opinion: Routinely checking for EPI in patients with chronic diarrhea, using fecal elastase, is unreliable in the absence of testing a formed stool*

A multitude of tests for EPI have been developed over the past several decades and classified as direct versus indirect measures of exocrine pancreatic function. However, many of these have poor sensitivity or specificity (e.g. serum trypsin levels, qualitative stool fat) and/or are available at only limited centers such as with the ^13^C mixed triglyceride (^13^C-MTG) breath test.

### 72*-*hour fecal fat test

The gold standard has been the 72-hour stool collection while the patient consumes a diet containing 100 g of fat per day. Fat malabsorption is diagnosed at > 7 g of fat per 100 g of stool per day, with severe steatorrhea at ≥ 15 g per day [18]. Unfortunately, this test is time-consuming and not easily tolerated due to bloating, abdominal discomfort, flatulence, and worsening steatorrhea. Additionally, errors can occur in stool collections and recording of fat intake [42]. Diseases that impact mucosal fatty acid uptake, such as Crohn’s disease, bacterial overgrowth, and short bowel syndrome, can cause abnormal values despite normal exocrine pancreatic function. However, the 72-hour stool collection has served to measure the effectiveness of PERT in EPI [43] for United States Food and Drug Administration (FDA) approval of PERTs.

### Fecal elastase test

The pancreas produces pancreatic elastase 1, which is a highly stable enzyme during intestinal transit [44]. This proteolytic enzyme can be measured in a fecal sample by an enzyme-linked immunosorbent assay [45, 46]. Because pancreatic elastase is highly stable during intestinal transit, the fecal concentration correlates well with exocrine pancreatic secretion [45]. Diagnostic testing using fecal elastase has some advantages over other tests because it does not require a timed stool collection or special diet, has a high negative predictive value, and has a high sensitivity in moderate to severe EPI when formed stools are analyzed [8, 42, 47, 48]. The reference range of less than 200 μg/g feces can be applied to both children and adults for the diagnosis of EPI [44, 45, 49]. Some consider values less than 100 μg/g feces as diagnostic of EPI, with fecal elastase values between 100 and 200 μg/g to be indeterminate and difficult to interpret [50].

In mild to moderate EPI, diagnostic testing using fecal elastase has a lower sensitivity (as low as 30%) and specificity, possibly resulting in an underestimation of EPI [45, 51–53]. In childhood, however, fecal elastase is a useful noninvasive screening test for EPI, demonstrating a negative predictive value of 99% for ruling out EPI [42]. Since fecal elastase is measured as a concentration in stool, watery stools will almost invariably result in low elastase values being measured and thus this non-invasive, pancreatic function test should be performed in a clinical setting where EPI is suspected and a formed stool can be analyzed. This has replaced the more cumbersome 72-hour fecal fat test. In addition, PERTs do not have to be stopped for fecal elastase testing since the porcine enzymes do not cross react with the human fecal elastase antibody.

## Treatment of exocrine pancreatic insufficiency

*Myth: PERT should be started at the lowest dose available and taken any time before a meal and at bedtime*

*Expert opinion: Titrate the dose of PERT to the presumptive degree of PERT and administer PERT with the first bite of a meal, and consider adding extra enzymes during or towards the end of the meal*

### Dietary management and lifestyle changes

Fat malabsorption is the predominant cause of the symptoms of pancreatic steatorrhea resulting in weight loss as well as deficiencies of fat-soluble vitamins A, D, E, and K. In patients with chronic pancreatitis, a low fat diet has been the recommendation in order to minimize the pain of this disease and, in conjunction with PERT, to effectively treat steatorrhea. However, in patients with CF, a high fat diet in conjunction with increased amounts of PERTs has been shown to improve the associated CF lung disease and thus low fat diets are no longer advocated in this disease. Fat soluble vitamins A, D, E, and K should be supplemented if indicated, and taken with PERT [54]. Consulting a dietitian is helpful to assess nutritional adequacy [55]. In addition, smoking has been proven to be a risk factor in acute pancreatitis, chronic pancreatitis, pancreatic cancer [56], and to be associated with reduced exocrine pancreatic function [57]. Therefore, smoking and alcohol cessation is recommended in EPI due to chronic pancreatitis.

### Pancreatic enzyme replacement therapy

The elimination of malabsorption, reduction of maldigestion-related symptoms, and the prevention of malnutrition-related morbidity and mortality is the goal for PERT [55]. This is most evident in CF, where prior to the availability of PERT, infants died within the first year of life. Prior to 2010, pancreatic enzymes were not FDA regulated and had variable consistency of activity. As a result, in 2010, the FDA mandated approval of all prescribed formulations of PERT. It should be noted that the clinical trials were relatively small (less than 40 subjects) and tested in subjects who were known to respond to PERTs. All pancreatic enzyme preparations are extracts from porcine pancreas (pancrelipase) and are available in preparations encapsulated in mini-microspheres or microtablets, which vary in particle size and pH-related release kinetics [58]. Enteric-coated pancreatic microspheres are designed to be acid resistant and pH-sensitive to protect lipase from denaturation by gastric acid. Unfortunately, confusion has arisen due to the many different dosage strengths of PERTs [59] (Table 1).

**Table 1:** Current Food and Drug Administration (FDA) approved pancreatic enzyme replacement therapies (PERTs)

Brand	Units of lipase
Creon	3000; 6000; 12,000; 24,000; 36,000
Zenpep	3000; 5000; 10,000; 15,000; 20,000; 25,000
Pancreaze	4200; 10,500; 16,800; 21,000
Ultresa	13,800; 20,700; 23,000
Viokase	10,440; 20,880 (requires acid suppression)
Pertzye	8000; 16,000

Enteric-coated pancreatic enzymes are most effective at a pH > 6. However, in patients with CF, the duodenal pH is < 6 [60]. The use of acid-suppression medications can increase gastric pH levels and theoretically improve the efficacy of PERT and decrease EPI symptoms [61, 62]. Current data may suggest a trial of acid blockers in patients with CF who have refractory steatorrhea [61–65]. However, a recent retrospective study demonstrated no improvement of the coefficient of fat absorption (72-hour fecal fat test) when using a proton pump inhibitor in pediatric patients with CF [66].

Uncoated exogenous pancreatic enzymes, such as Viokase (Aptalis Pharma), are thought to mix well with intragastric nutrients and rapidly release high duodenal lipase amounts for fat digestion [58]. The addition of acid-suppression medications is required to prevent degradation of non-enteric coated pancreatic enzymes [58]. Only non-enteric pancreatic enzymes have been shown to improve the pain in a subset of patients with chronic pancreatitis. The use of unprotected exogenous enzymes in combination with enteric-coated enzymes has previously been recommended for the treatment of refractory EPI [58, 67]; however, Kalnins et al. [68] showed no improvement in nutrient digestion (fecal fat, energy, and nitrogen output) when unprotected pancreatic enzymes were added to the conventional enteric-coated enzymes in 14 pediatric patients with CF.

### Dosing and frequency of PERT administration

Dosing and frequency of administration are difficult aspects of PERT treatment since different enteric-coated microspheres are not bioequivalent in vitro [69–71], and there are not enough clinical studies between preparations to define in vivo bioavailability. In these in vitro studies, the preparations varied in dissolution time (49–71 min half-life time) and in optimum pH (pH 5.0–5.8).

Several countries have recommended different doses of PERT. The Australasian Pancreatic Club [72], The Italian Association for the Study of the Pancreas [8], and The Spanish Pancreatic Club [73] recommend 25,000–50,000 lipase units per main meal in adults. Unfortunately, the evidence for these recommendations is relatively weak as emphasized by The Australasian Pancreatic Club in their recent study on the management of pancreatic exocrine insufficiency [72]. In addition, a study from the Netherlands by Sikkens et al. [74] found that 70% (*n* = 161) of the patients with EPI caused by chronic pancreatitis were under-treated and reported steatorrhea-related symptoms, despite PERT (median enzyme intake of 6 capsules, 25,000 lipase units per day). These differences in recommendations demonstrate the significant confusion over dosing and administration amongst medical practitioners.

Likewise, there is no consensus over frequency of PERT administration. In 1977, DiMagno et al. [75] demonstrated that administration of PERT during a meal was as effective as hourly administration over the day to decrease steatorrhea. Other recommendations based on several reviews are to take 50% of the exogenous pancreatic enzymes at the beginning of the meal and 50% half-way through [76], pancreatic enzymes during or immediately following the meal [77], or lastly, 25% of the enzymes with the first bite, 50% during the meal, and 25% with the last bite [78]. In addition, a recent randomized three-way crossover study of 24 patients using 40,000 lipase units per meal compared three different administration schedules with PERT before meals, during meals, or after meals using the ^13^C-MTG breath test to measure fat absorption [79]. The percentage of patients who normalized fat digestion was 50%, 54%, and 63%, respectively. Thus, no statistically significant differences were found between different administration schedules, however, they did recommend giving PERT during or after meals.

In a patient with suspected EPI with a known history of pancreatic disease, empiric therapy with PERTs may be indicated without formal testing. A clear response would be both diagnostic for EPI as well as therapeutic. In addition, if there is a poor response to PERT, one should consider concurrent gastrointestinal comorbidities such as lactose intolerance, enteric bacterial infection, parasites (especially giardia), small intestinal bacterial overgrowth, biliary disease (cholestasis), colitis, celiac disease, short bowel syndrome, and Crohn’s disease [80]. Other reasons could be insufficient dosing, lack of compliance, inadequate timing of PERT administration, and poor diet (Table 2).

**Table 2:** Treatment strategies for lack of response to pancreatic enzyme replacement therapy (PERT)

Treatment strategies	
• Increase dosage	
• Check compliance with the patient	
• Add acid inhibitor	
• Consider adding enzymes during and towards end of meal	
• Consider microspheres, possibly adding a rapid release enzyme preparation	
• Look for evidence of concurrent gastrointestinal disorder	

PERT should be taken with the first bite of a meal and consider adding extra enzymes during or towards the end of the meal. Thus, if consumption of a meal is less than 15 min, all enzymes can be taken at the beginning of the meal; for a 15- to 30-minute meal, we suggest taking half the enzyme capsules with the first bite and the other half in the middle of the meal; for more than 30 minutes, we recommend taking one third at the beginning, one third in the middle and one third at the end. The rationale for taking pancreatic enzymes throughout the meal is to mimic the action of our own endogenous pancreatic enzymes, where secretion from the gland occurs throughout a meal. Specifically, the more food that is ingested and/or the grater the amount of fat in the diet, the higher the amount of endogenous pancreatic enzyme secretion; thus, the number of PERT capsules consumed should reflect this. Table 3 gives a suggested clinical overview of PERT dosing for different age groups [17, 19, 80].

**Table 3:** Pancreatic enzyme replacement therapy (PERT) suggested dosing in different age groups

Age group	Units of lipase
Infant	2000–4000 units/120 mL formula or breastmilk
Child age < 4 years	1000 units/kg/meal 500 units/kg/snack
Child age ≥ 4 years	500 units/kg/meal 250 units/kg/snack
Adult starting dose	50,000 units/meal 25,000 units/snack
Adult maximum dose	150,000 units/meal 70,000 units/snack

## Summary

Since the required FDA approval of PERT in 2010, we now have reliable medications for the treatment of EPI. These therapies have improved signs and symptoms related to EPI such as steatorrhea and abdominal discomfort, weight loss, malnutrition, and possibly even quality of life. PERT is lifesaving in CF, since prior to PERT, babies with CF died of malnutrition within the first year of life. However, there is still much confusion amongst medical practitioners over the best diagnostic approach as well as dosing and administration of PERT. Many countries have developed different guidelines regarding dosing and administration of PERT, calling out for a consensus and a practical guide for the diagnosis and treatment of EPI using exogenous pancreatic enzymes. In addition, evidence supports that patients are being undertreated with PERT and may potentially benefit from more adequate therapy.

There are two areas that need emphasis. First, physicians need to know when to test for EPI and agree on using the same methods for diagnosing EPI. Over the last decade, there has been a change in diagnostic approach for EPI from the unreliable qualitative stool test and the cumbersome 72-hour fecal fat collection, to the more sensitive, but less specific, fecal elastase test, especially in patients with mild to moderate EPI. Many physicians do not realize the need to have formed stools analyzed and thus, in chronic diarrhea, this may be problematic. Ultimately, what is critical is the early diagnosis and optimization of treatment of EPI.

Second, there must be optimization of the currently available therapies for EPI. The existing studies vary on recommendations for dosing of exogenous pancreatic enzymes ranging from 25,000 to 80,000 lipase units per main meal, and there is uncertainty about administration of PERT before, during, or after the meal. In addition, the treatment goals differ from reducing pancreatic steatorrhea and elimination of maldigestion and malabsorption, to the prevention of malnutrition-related morbidity and mortality. This is all complicated by the myriad of pancreatic enzyme formulations at a wide array of dosing strengths. Thus, it is not surprising that confusion amongst physicians exists over the optimal dosage, administration schedule, and what to aim for in PERT.

Our recommendations for PERT are: (1) titrate the dose of PERT to the presumptive degree of PERT, (2) administer PERT with the first bite of a meal and consider adding extra enzymes during or towards the end of the meal, (3) consider using microspheres, possibly adding a rapid release enzyme preparation and/or acid-blockade, and (4) adjusting the dose to the fat content of the meal.

## Abbreviations

^13^C-MTG: ^13^C-mixed triglyceride
CF: cystic fibrosis
CFTR: cystic fibrosis transmembrane conductance regulator
EPI: exocrine pancreatic insufficiency
FDA: Food and Drug Administration
PERT: pancreatic enzyme replacement therapy
PRSS1: cationic trypsinogen
SPINK1: serine peptidase inhibitor Kazal Type 1
